# Experienced consequences of being diagnosed with ADHD as an adult – a qualitative study

**DOI:** 10.1186/s12888-015-0410-4

**Published:** 2015-02-26

**Authors:** Sara Lina Hansson Halleröd, Henrik Anckarsäter, Maria Råstam, Marianne Hansson Scherman

**Affiliations:** Department of Clinical Sciences, Lund University, Malmö, Sweden; Centre for Ethics, Law and Mental health (CELAM), Institute of Neuroscience and Physiology, University of Gothenburg, Gothenburg, Sweden; Department of Clinical Sciences, Lund University, Lund, Sweden

**Keywords:** ADHD, Adult, Diagnosis, Patient experiences, Phenomenography, Qualitative research

## Abstract

**Background:**

Despite increasing knowledge of attention deficit hyperactivity disorder (ADHD) across the life span, there is still little research on adults’ own experiences of being diagnosed with ADHD. The aim of the present study was to explore and describe patients’ experiences and perceptions of being diagnosed with ADHD in adulthood. The study can be seen as an attempt to validate the diagnosis from a patient perspective.

**Methods:**

Twenty-one adults diagnosed with ADHD were individually interviewed. The interviews were open-ended and exploratory, analysed with a qualitative phenomenographical approach, and the results were described in categories.

**Results:**

Positive experiences were dominant, but there was a complex intra- and inter-individual variation of experiences. Descriptions focused on the diagnosis, on identity, and on life. The diagnosis was described as explaining a previously inexplicable life history, but was also questioned, both as a phenomenon and in relation to the individual (*the diagnosis* in focus). It was experienced as providing self-knowledge and increased value, but could also cause devaluation and concern about identity (*identity* in focus). It meant help to achieve a better life, but was also perceived to restrict possibilities and cause disappointment over lack of professional help. It could lead to a wish for an earlier diagnosis that could have spared suffering, as well as to a changed view of the participants’ relatives (*life* in focus). All but one of the interviewees expressed important positive consequences of being diagnosed with ADHD. About half of them acknowledged negative aspects of being diagnosed, but none regretted going through the neuropsychiatric evaluation.

**Conclusions:**

From a patient perspective, there are major positive consequences of being diagnosed with ADHD, compared to the undiagnosed situation. Knowledge of the individual’s combination of experiences is important for professionals, as these experiences can affect well-being and interfere with treatment. Negative experiences in particular might need to be addressed in the treatment work.

## Background

Attention deficit hyperactivity disorder (ADHD) is one of the most common childhood-onset psychiatric disorders, with an estimated prevalence of 5-7% in children and adolescents (e.g. [1,2]). It appears to persist into adulthood in about two thirds of individuals, either as the full diagnosis (15%) or in partial remission (50%) [3]. Prevalence rates of 2.5% [4], 3.4% [5], and 5% [2] have been suggested for ADHD in adults.

Estimating the prevalence of adult ADHD is difficult because the DSM clinical criteria have not been validated for the adult population [6] and the clinical presentation of ADHD tends to change during adolescence. Hyperactivity and impulsivity usually become less obvious and are expressed differently, for example as an inner restlessness, inability to relax, and a pattern of starting new jobs and relationships on impulse [7]. Problems related to inattention can become painfully impairing as demands become more complex and the individual is expected to function more independently [8], and often manifest as symptoms including disorganization, boredom, and sensitivity to stress [7]. Many individuals experience mood lability and short-fused temper outbursts [9]. Comorbid disorders are common, including substance use, mood, anxiety, personality disorders, and other neurodevelopmental disorders, and can sometimes dominate the picture [10-12]. Good coping strategies to overcome ADHD symptoms, adaptation of occupational environments, and supportive family structures can further mask impairments [13]. Still, these individuals frequently struggle with impairments in every-day life, academic, and social functioning [14,15] as a result of the condition, significantly affecting quality of life [16]. Several studies have described the burden of living with ADHD (e.g. [13,15,17-19]) and undiagnosed ADHD (e.g. [20-22]). Studies suggest that early diagnosis can improve quality of life, self-esteem, overall functioning, outcome, and long-term prognosis [16,23-26]. In contrast, Travell and Visser [27] question the value and validity of diagnosing young people, warn about negative psychological effects and argue that the voice of the young person should be heard and taken into account.

While there is increasing knowledge of how ADHD can affect individuals across the life span, qualitative studies exploring the affected adults’ own experiences and perceptions of being diagnosed with ADHD are still sparse. From previous qualitative studies concerning different aspects of ADHD, it appears that being diagnosed with ADHD has several positive consequences: understanding of own difficulties [28-30], reduction of self-blame/improvement of self-perception [28-30] and improved functioning/coping [27-31]. The main negative effect described in the previous studies was experiences of stigmatization [27,29-31]. However, none of these studies provides a systematic coverage of the variation of positive and negative experiences of being diagnosed with ADHD and the meaning of these, as they do not primarily and solely focus on the whole and specific phenomenon of receiving a *diagnosis* of ADHD. Instead, they target certain aspects of the phenomenon, such as effects on coping, or related phenomena, such as symptoms. Some of the studies have assessed younger populations [27,31].

The aim of the present study was to explore and describe qualitatively different ways in which adults perceive and experience being diagnosed with ADHD. We used a phenomenographical method to discover a wide variation of experiences that can be used in a clinical context. In addition, the study can be seen as an attempt to validate the ADHD diagnosis from the patients’ perspective. For this purpose, we find it important to isolate the phenomenon (being diagnosed with ADHD), so that the found variation concerns the same phenomenon and not a variation of different phenomena. In addition, the aspects included in the phenomenon were not predefined, but determined by the participants. Even though it is impossible for researchers to be completely unaffected by their own perspectives in data collection and analysis, the intention was to “put these within brackets” in order to come as close as possible to understanding the diagnosis as the patient does. The knowledge gained from this study is therefore relevant for the way we diagnose and care for these persons, but could also contribute to the debate concerning the validity of the ADHD diagnosis in adults.

## Methods

### Participants

All twenty-three individuals who were assigned a diagnosis of ADHD at the Psychiatric Outpatient Clinic in Mölndal, Sahlgrenska University Hospital, Unit for Autism and ADHD, during the research period, and eligible for the study, were consecutively asked to participate in the study. None had been diagnosed with ADHD before. The authors involved in data collection and analysis had no previous connection to the clinic and its patients. Of those invited, 21 (11 women and 10 men) agreed to participate. One man declined because he felt that being diagnosed with ADHD was “the worst thing he had ever experienced” and another because he was not feeling well. The patients had not received any treatment at the clinic, but had been referred elsewhere. The study targeted individuals 20-35 years of age, as this was the prioritized age group of the clinic. Four individuals outside this age span were included (38, 38, 52, and 57 years respectively), because they were patients at the clinic during the research period, resulting in a mean age of 32.2 years (SD 9.2, median 31). Time since diagnosis varied between 3 and 15 months with one outlier (27 months, mean 7.5, SD 3.9, median 9). Individuals with other psychiatric disorders or conditions significantly affecting functioning, behaviour, or life situation (intellectual disability, psychotic disorder, bipolar disorder, autism spectrum disorder, personality disorder, ongoing depression, diagnosed brain lesion, ongoing substance use disorder, or criminality) were not eligible.

### The phenomenographical approach

We chose a qualitative research method because our research interest was not verification of a predetermined idea, but exploration and discovery that could lead to new insights into, or a deeper understanding of, having been through a neuropsychiatric evaluation and assigned a specific diagnosis – ADHD. Qualitative studies begin with vaguely formulated research questions where, ideally, nothing is predefined or taken for granted [32-34]. The phenomenographical method [35] was chosen because, in contrast to other qualitative research approaches, it aims to discover the variation of qualitatively different ways of experiencing a phenomenon, to describe these in categories, and to use the discovered variation for educational purposes in accordance with “the variation theory of learning” which was developed from the phenomenographical research method [36]. In this case, the patient experiences are intended to be used in clinical situations, which could be seen as learning situations.

### Procedure

The participants were initially included by the staff at the clinic (supervised by MR) and then contacted by the interviewer (MHS, experienced in qualitative interviewing and research) about one week after having consented to participate. The interviews took place in the participants’ homes (with two exceptions where the individuals preferred other settings), as the contextual conditions will influence what is talked about [37] and a “clinical” setting would possibly have biased the results towards a more “medical” narrative. After a short introduction, the opening question was: “When you think about having ADHD, what comes to mind?” Other questions concerned areas such as possible changes in daily life or in their relationships with themselves and others, possible experiences of gains or losses, and feelings and thoughts related to being diagnosed. Finally, the participants were asked if, given the knowledge and experiences they had at the time of the interview, they would have chosen to go through the neuropsychiatric evaluation.

The interviews were open-ended and exploratory within the phenomenon and conducted in an informal conversational manner, which allowed the interviewees to talk freely and to express their own experiences of being diagnosed with ADHD. The interviewer attempted to go beyond a surface level where the person being interviewed says no more than what might be anticipated. The interviewer tried to follow the way in which each individual reasoned, meaning that the participants did not necessarily talk about the same aspects of the phenomenon, as each spoke from their own life situation. Provocative questions, follow-up questions, and prompts were used with the aim of going more deeply into the matter in hand. The ambition was to let the participants concretize their experiences. The interviews lasted an average of 1 hour, and were tape recorded and transcribed verbatim. The interview stage was concluded when a rich range of different experiences had become apparent, experiences were beginning to recur in new interviews, and no new experiences appeared in new interviews.

### Data analysis

Interview transcripts constituted the data for the analysis, following the phenomenographical method originally outlined by Marton [38] and later described in detail by Dahlgren and Fallsberg [39]. The analysis took place in seven steps. In the first step, *familiarization*, all the interviews were read thoroughly in order to get acquainted with the material and to obtain an overview. Next, the *condensation* process consisted of selection of utterances, from all the interviews, relevant to the research question: experiences of being diagnosed with ADHD. The meaning of an utterance was interpreted in relation to the context from which it was selected. These selected quotations made up the data pool for further analysis. They were *compared* concerning similarities and differences, and excerpts with similar meaning were *grouped* together in categories. The *articulation* step constituted a preliminary description of the essence of the similarity within each group of statements. The categories were *labelled* according to the essence of their meaning. In the final step, *contrasting*, categories were compared regarding similarities and differences, to reach a definite description of the unique character of each category and a higher level of abstraction. There was a constant interplay in the entire process between the various steps of the analysis. The validity of the analysis was secured through dialogical intersubjectivity [40]; that is, the interviews were first read and analysed separately by two of the authors (SLHH and MHS), and the results were then compared for similarities and differences. Definitions of categories were discussed and tested against the data, adjusted, re-tested, and adjusted again. The material was discussed until agreement was reached and the results appeared stable.

### Ethical considerations

The study was approved by the ethical review board at the University of Gothenburg (Dnr: 047-07). All participants gave written informed consent.

## Results

The phenomenographical analysis resulted in an outcome of eleven main categories, four of which consisted of two sub-categories each. These categories and sub-categories described the patients’ different experiences, which were also, to various degrees, value judgements of having been assigned an ADHD diagnosis. The interviewees welcomed the ADHD diagnosis as an explanation for their problems and for the experience of being different (category 1A), but ADHD was also perceived to be associated with certain positive traits (category 1B). On the other hand, different reasons for doubting or questioning the diagnosis were expressed (category 2). The diagnosis was experienced to bring new knowledge (category 3), to affect identity in a positive (category 4) and/or negative way (category 5), and/or to create a feeling of insecurity concerning identity (category 6). The diagnosis was described to improve (category 7) or impair (category 8) the possibilities of having a good – normal – life. There were also experiences of having been left without the expected help after having been assigned the diagnosis (9). Experienced improvement of life conditions after diagnosis could cause sadness/anger over the lost years prior to diagnosis and a wish for earlier diagnosis (category 10), an eagerness to detect ADHD symptoms early in their own children, and grief over older relatives having gone through life undiagnosed and thus without help for their difficulties (11).

Contrasting the categories in the last step of the analysis revealed that the categories concerned how the individuals related to the *diagnosis* (categories 1-2), and how the diagnosis affected *identity* (categories 3-6) and *life* (categories 7-11). Identity is used here in the sense of what defines a unique human being in their own eyes or in those of others. Life is used in the sense of existing, being. Table 1 presents the focus of each category on diagnosis, identity, or life; the positive or negative value judgement of each category in relation to being diagnosed; and the distribution of interviewees between the different categories.

**Table 1:** **Distribution of interviewees across different categories in the three focus areas: diagnosis, identity, and life**

**Interviewee**																						
**Category**	**1**	**2**	**3**	**4**	**5**	**6**	**7**	**8**	**9**	**10**	**11**	**12**	**13**	**14**	**15**	**16**	**17**	**18**	**19**	**20**	**21**	**Total number of interviewees**
*Diagnosis*																						
1. The diagnosis is an explanation +																						
A. for difficulties	x	x	x	x	x	x	x	x		x	x	x	x	x	x	x	x		x	x	x	19
B. for positive traits	x		x												x	x				x		5
2. I question or have doubts about the diagnosis (-)					x		x				x							x		x		5
*Identity*																						
3. I get to know myself better through the diagnosis +/-	x	x	x	x		x				x	x		x			x	x	x		x		12
4. The diagnosis gives me an increased value +																						
A. I value myself more highly	x	x	x	x		x	x	x		x	x		x	x	x	x	x		x	x	x	17
B. Others don’t think as badly of me anymore		x	x		x		x	x		x		x	x	x	x		x		x	x	x	14
5. The diagnosis causes decreased value -	x			x	x	x		x			x			x	x			x		x		10
6. The diagnosis raises questions concerning who I am (-)	x					x					x											3
*Life*																						
7. The diagnosis implies help to achieve a better life +																						
A. I can get help from others	x	x	x	x	x	x	x	x	x	x	x	x	x	x	x	x	x		x	x	x	20
B. I can help myself	x		x				x	x			x		x	x	x		x					9
8. The diagnosis limits my possibilities -																						
A. Fear of discrimination	x		x								x			x	x			x				6
B. The diagnosis as a self-fulfilling prophecy											x									x		2
9. I did not get the follow-up and help I expected (-)				x							x	x			x	x						5
10. I wish I had been diagnosed earlier in life +			x		x	x		x	x	x		x	x		x	x	x		x			12
11. Being aware that ADHD is hereditary makes me see my parents and children differently +	x		x							x				x		x						5
Total number of categories	10	5	10	6	6	7	6	7	2	7	11	5	7	8	10	8	7	4	5	9	4	

The +/(+) and -/(-) signs indicate whether each category is positively/slightly positively or negatively/slightly negatively charged in relation to being diagnosed.

All of the interviewees expressed experiences belonging to more than one category (range: 2-11 of the 15 categories/sub-categories). There was a variation *between* individuals as well as *within* each individual in the way that they experienced being diagnosed with ADHD, and the individual experience could be complex. Sometimes, experiences that may seem incompatible or illogical were expressed by a single individual. For example, the same person might describe how the diagnosis of ADHD had had both positive and negative effects on identity, and had created both possibilities and obstacles in life. A person could express that the diagnosis explained their difficulties, yet still have doubts about it.

All but one of the interviewees (20/21) expressed major positive consequences of being diagnosed with ADHD, mainly that the diagnosis was connected with help towards a better life (20/21), that it increased their value (19/21), and/or that it improved their understanding of themselves and their history (19/21). About half of them (12/21) described disappointment that they had not been diagnosed earlier in life. Negative consequences of being assigned a diagnosis of ADHD were mainly expressed through experiences of decreased value (10/21) and/or through fear of restricted possibilities in life (7/21). Even though about half of the individuals acknowledged some of these negative aspects, none, when asked directly, regretted going through the neuropsychiatric evaluation; all would have chosen to go through it again if they were still undiagnosed at the time of the interview. A minority (5/21) described various degrees of doubt in relation to diagnosis.

Each category is described in greater detail below, and is illustrated by interview quotations. The numbers within parentheses after the excerpts represent the code numbers of the interviewees. Ellipses (…) mark parts of conversation which have been omitted, and words within brackets are clarifications made by the authors. The abbreviations “IP” and “I” represent the interviewed person and the interviewer respectively.

### Diagnosis

#### The diagnosis is an explanation

##### An explanation for difficulties

The diagnosis of ADHD provided a satisfying explanation for recurrent problems, difficulties, shortcomings, and failures experienced in school, at work, or socially: “It was the answer to many years of wondering” (16). Being diagnosed could be accompanied by a feeling of relief and gratitude: “I’m grateful that I got it [the diagnosis] in the end … I’ve been searching for my entire life and trusted that someone would finally understand that something isn’t right” (10). The interviewees no longer needed to put energy into trying to find explanations for the feeling of being different and for unsuccessful behaviours or negative events that were previously difficult to understand: “It was a relief to find out what was wrong, why I got into fights, why I always quit my job, why I always get tired of people, and why I always get so pissed off at everyone” (6). Their life histories were given a new meaning: “I can filter myself and the world around me through it [the diagnosis] … I can explain why things happened before or I can explain what may happen after” (1).

##### An explanation for positive traits

Some positive traits were attributed to ADHD and perceived as resources, such as creativity, new thinking, passion, quickness and productivity: “I see ADHD as something positive, almost more positive than negative. I wouldn’t have been able to get everything done if I hadn’t had ADHD … Everyone could probably use a bit of ADHD at some point in their lives to clear things up” (15). ADHD could contribute to an interesting and exciting life: “If I could choose a life without ADHD, I wouldn’t choose it, I would like to have it … you get to have a pretty fun life, I think … there’s a lot happening all the time with me” (1). A person with ADHD was described as being spontaneous, funny, and “a little crazy” in a positive way.

#### I question or have doubts about the diagnosis

There were different degrees of doubt concerning the ADHD diagnosis, and various reasons and arguments were expressed. Firstly, the actual existence of ADHD as a diagnosis was questioned. Some interviewees expressed the idea that modern society is not adapted for everybody, or that symptoms regarded as ADHD-related could instead be caused by adverse childhood conditions or by external factors in the society/environment: “These symptoms, I think they’ve also become an effect of the way we live” (18). The question of where to draw the diagnostic boundaries was raised.

Secondly, the interviewees had experienced questioning over the accuracy of their diagnosis, both by themselves and by others. One reason for this was that the individual did not seem to fit into the stereotypical conception of what a person with ADHD is like, or did not resemble other people with ADHD in their circle of acquaintances:

I’ve had discussions with people and I’ve said that I have ADHD, then I got the answer ‘really, you do? But you’re so calm, you never get angry’ … Many people believe that I don’t have ADHD … their picture of ADHD is something else … I don’t fit into their picture. (7)

If the individual had been diagnosed with other conditions prior to ADHD, the ADHD diagnosis could lose some of its credibility. Not responding to medication or responding well to non-medical therapy were other reasons for questioning the correctness of the diagnosis. Finally, the diagnosis could be perceived as unnecessary: “I don’t need to have a diagnosis to have problems with certain things … I work with what I have to work with, then whether or not it’s ADHD, it just isn’t so damned important to me” (18).

### Identity

#### I get to know myself better through the diagnosis

The focus of this category was on learning about oneself and one’s functioning: “You gain deeper insight into who you are, that’s what you really want, who am I, what am I doing?” (1). A new self-understanding developed: “You gain better self-awareness, because you aren’t always what you think you are” (3). New insights could change the understanding of how oneself and one’s behaviour were experienced by others:

Then I was sad because my behaviour has been a little special, since I’ve been extremely cocky … so then I started thinking about it, hell they think I’m a fool, not that I have ADHD, but just purely for my behaviour, that I always look like I’m not listening. (1)

When certain characteristics and behaviours were connected to ADHD, they could be revised and perceived as wrong and unwanted:

Everything, like half my life, all I’ve done, all the feelings I’ve had, might not have been so right … that I’ve been false in some way, that it wasn’t right to think this way, and act this way … you do things wrong, everyone who criticized you, suddenly it feels like they were right, that I have fought against them and tried to do things my way. (6)

When problems and difficulties were seen as symptoms of a diagnosis, they could be painfully transformed from temporary to lifelong: “Now I have this shit, I thought, because I knew there was no way to get better, there are no tricks to become healthy … so it’s a handicap … I have to live with it” (1).

#### The diagnosis gives me an increased value

Being diagnosed with ADHD was described by the participants as legitimizing difficulties and unacceptable behaviours, thus improving their self-worth as well as other people’s views of them.

##### I value myself more highly

The interviewees perceived the ADHD diagnosis as improving their value and creating a more positive self-conception. Having ADHD was less harmful to identity than being characterized as “stupid”, “lazy”, “angry”, “bad tempered”, “aggressive”, or “crazy”:

You don’t feel nearly as stupid any more … I don’t have to be ashamed any more, I know it’s a disease, I can’t do anything about it, it isn’t my fault, it’s hereditary, before it was that you were careless and lazy. (15)

The diagnosis could reduce the feeling of guilt, as the individual became less responsible for failures and misbehaviours: “It isn’t my fault that I’m a bad person in some cases” (1). Even the experience of being disabled and abnormal could be reduced: “Before I didn’t know why I was like that, and then I felt more abnormal … now I feel more normal, loads of people have ADHD” (14). The diagnosis seemed to be separated from the self: “There was nothing wrong with me … I just had a diagnosis” (20).

The experience of increased value meant treating themselves kindly and not tolerating bad treatment from others: “I’ve probably become tougher, I think … now I know why they can’t get on my case … I don’t have to take any crap” (7). Individuals respected their limitations: “Before, it was more that I wanted to be normal and keep up with others, now it’s more that I’m normal, it’s just too much for me … why do something I can’t manage?” (14). They reduced their demands on themselves and judged themselves less harshly:

I felt that I shouldn’t set such high standards for myself, but I would accept that I’m the way I am, and I wouldn’t come down on myself and think ‘you’re an idiot, everyone can do this’, yes, everyone who has that ability, but I don’t have that ability. (13)

##### Others don’t think as badly of me anymore

The interviewees, within this category, experienced their ADHD diagnosis as making it easier to communicate their problems to others: “When I didn’t know what it was, I didn’t know how to explain it either” (7). When an individual’s shortcomings were interpreted in terms of ADHD, they became legitimate and other people’s understanding of them changed, resulting in a changed attitude and a kinder treatment: “They excuse me, they think ‘she isn’t crazy … she just has ADHD’” (20). It was experienced as less disparaging to have ADHD than to be categorized as, for example, “a disruptive child”, “impolite”, or “nonchalant”: “I’d rather be stamped with the ADHD diagnosis than walk around and have people think I’m weird” (12) and “It’s stranger to be weird or different or a difficult child than to have a diagnosis” (16). After they were diagnosed, people did not think as badly of them as before, and irritation and demands were reduced: “People treat me very differently now, a huge difference … I’ve had contact with social services for many years … I’ve felt like they were treating me as though I’ve been lazy and not wanting to achieve anything, and now they’ve completely changed their tone” (7). Instead of being “punished”, adjustments were made and they were shown consideration and indulgence: “Finally they understand what it’s like for me, and society can accept me for who I am” (3). The interviewees no longer had the same need to lie, cheat, or find excuses to avoid or cover up failures and difficulties, in order to preserve their value in relation to others: “Nobody wants to say ‘I’m worthless’, and that’s why I’ve been forced to cheat in school many times … if they’d just given me an extra five minutes, I would have managed it” (3).

#### The diagnosis causes decreased value

Within this category, the ADHD diagnosis was experienced as decreasing a person’s value: “You’re worth a little less than other people [when you have ADHD]” (18). The experience of devaluation could spring from the comparison of oneself to others: “I want to be like everyone else” (4) and “I don’t want to be worse than others … there’s something wrong with me … I don’t work like the others” (11). It could also be the result of stigmatization, in the sense of having a characteristic which makes a person differ from the norm in a certain context, resulting in devaluation [41]:

I don’t say ADHD, I say that I have dyslexia, ADHD sounds so ugly in everyday speech … I heard adults who were talking about it yesterday … then they said ‘yes, it’s typically the damn kids with ADHD’ … My daughter doesn’t know it, ADHD is still very ugly at their age. They bully each other on the playground and ‘fucking DAMP kid’ is a term that is used a lot. (15)

The devaluation was described in terms of being, for example, “different”, “stupid”, or “less capable”: “When I’m a bit depressed I sometimes think it feels a little like a defect” (8). The diagnosis was associated with “shame” and “low status”, and with being “judged” and “disrespected”:

You become a different person in the eyes of others, you might not be respected for who you are and your thoughts and opinions are not valued as highly as before … She checks the situation all the time as though there is something sick about me, that I’m someone weird … (6)

The interviewees described feeling excluded, like an outsider; not belonging to the “healthy world”, but to an outgroup: “Now it’s like I have this diagnosis, OK now I get to join this club” (11). Being in need of help and receiving special treatment could in itself be stigmatizing:

I didn’t want special treatment in front of others and in front of myself … Just because I have a diagnosis, it means you should have extra help because you can’t manage things like all the others manage and then you feel a bit handicapped. (20)

Sometimes the interviewees did not reveal their diagnosis to others because they were afraid of being looked down on: “I don’t tell anyone, when you’re an adult you don’t want to seem too stupid” (5). One approach to reduce the negative effect on identity was to point out that they only fulfilled some of the ADHD criteria: “I say that I have ADHD and that I have only two things in it … they shouldn’t think that I have everything” (5).

#### The diagnosis raises questions concerning who I am

Some interviewees spoke about the possibility that the ADHD diagnosis might interfere with identity, raising questions and worries concerning identity. Concern was expressed as to whether one could remain the same person after having been assigned a diagnosis of ADHD: “Do I have to start all over again or will I become someone else now, I was confused” (6). There were worries about how to retain one’s individuality and identity, both in relation to oneself: “It’s easy for me to over-interpret things through the lens of ADHD … it’s like I identify with this” (11) and in relation to others: “I’m still me … I don’t want to be seen as an ADHD person, I want to be seen as [says her name] …” (11).

Another issue was how much of an individual’s characteristics and behaviour could be explained by ADHD: “How much of me is ADHD, of what I do? … Do I have ADHD only when I get angry or do I have ADHD all the time?” (6).

### Life

#### The diagnosis implies help to achieve a better life

Being in need of help could serve as an important motivating factor for wanting a neuropsychiatric evaluation: “First I had an evaluation made that I ignored … it felt silly to get some diagnosis … but then I realized that I need help … I have no life …” (8). The search for help prior to being diagnosed had sometimes been desperate: “I pretended to be an alcoholic because I was thinking, they get help, they get loads of help” (10). Individuals represented in this category felt that being diagnosed could improve the possibilities of having a good – normal – life. The knowledge of what they were dealing with made it possible to try to do something about it themselves or to seek professional help: “It’s hard to do anything about it if you don’t know what it’s all about” (8).

##### I can get help from others (including professional help)

The interviewees experienced their ADHD diagnosis as opening possibilities of receiving help from the people around them in daily life and from different institutions in society such as health and medical care, the social insurance office, the employment agency, and the social services: “A person who is just angry, they have no guidelines in the same way … but when I got a diagnosis, it was more, ‘oh great, so that’s how we should deal with it’” (14).

One consequence could be reduced suffering: “… chance for a normal or comfortable life instead of a life of torment … because it’s been a torment considering that my body is so restless” (9). Another effect was improved functioning: “The medicine works so that I can manage for a whole day and then I feel it’s far more likely I’ll be able to cope with a real job, and that’s really great” (17). Sometimes effects on the individual’s behaviour could be of vital importance: “It was untenable before … the medicine has made me grow up … it’s OK for a 20-year old to behave immaturely, but as a 30-year-old, it isn’t accepted …” (19).

The change in living conditions could be powerful. This was sometimes described as necessary or life-saving:

In my case it was a life, a life with dignity … The diagnosis and the medicine together and then suddenly doors are opening everywhere … I can finally do things I’ve longed to do … like read, knit and go to the gym … I have a positive feeling within me that life is beautiful. (10)

##### I can help myself

When the interviewees understood their own reactions in terms of ADHD, they knew what to expect from themselves, and were less likely to get angry with themselves when, for example, they got to the point of losing their concentration or their temper. Instead, they could focus their energy on managing the situation:

I couldn’t vacuum because the vacuum hose bending was enough to make me so angry that I started to break things. It was almost like things got better just from knowing that it was ADHD, because not knowing why I was so angry just built up my irritation. (14)

When they recognised a certain behaviour as having been caused by ADHD, they no longer defended it but instead regarded it as incorrect, motivating them to try to change it:

In the past, I could make an explanatory model based on others … like, I could have the perception that I’m a genius, the others are stupid … now I mainly think that there’s a risk of me being a burden to others … if there is any opportunity for me to improve my behaviour or who I am, obviously I do it. (1)

The interviewees described looking at themselves from a different perspective: “I feel like I’m constantly looking for kicks … you get a completely different picture of it … as an adult, I think either I have to replace it with something else or I quite simply have to shape up” (3). Their new understanding of their own difficulties could inspire a search for ways of handling their ADHD symptoms, resulting in different strategies: *avoiding* situations where they had to face major limitations related to ADHD, *compensating* (e.g. through the use of different planning techniques or preparing themselves before facing challenging situations), and *fighting* (e.g. to defy impulses or the feeling of restlessness):

I am impulsive and can just do an about-face … and then I might think ‘it’s just an impulse, it doesn’t have to be that way, it’s just the way it feels right now’ … I have to think a bit harder because I can easily become annoyed, angry at things or people and stuff and that may not always be the case if I think about things a little longer. (8)

This could improve their ability to handle different situations in daily life. One interviewee said that the knowledge of having ADHD had helped him become a better driver, and also a better parent through enabling him to read to his children:

… then you sit down properly and read, the end result will be positive. I know it’s hard, so then I bloody well have to do it … You have to deal with driving a car too … when it’s hard to stick to the speed limit, because it’s slow, I know why I think it’s slow, and then I might as well follow the speed limit. (1)

Another person described how the ADHD diagnosis made her avoid potentially harmful situations: “I would never dare to take drugs … because I know what it would certainly mean for me” (10).

The diagnosis also acted as a motivating factor through confirming pre-existing coping strategies:

I think the diagnosis made me more motivated to do it, because now I know what I’m doing is right, otherwise I would have done it at random and thought maybe it was right, but now I know it’s absolutely correct. (3)

#### The diagnosis limits my possibilities

There was some concern that an ADHD diagnosis might limit their possibilities in life, either through being an obstacle in relation to society or through the loss of self confidence.

##### Fear of discrimination

Some interviewees expressed the worry that the surrounding world, society, and different authorities could judge them differently and more negatively if they had a diagnosis of ADHD, resulting in other people distancing themselves from them, difficulties starting a relationship, or difficulties obtaining for example employment, a certain education, life insurance, or a driving licence. As a consequence of this, the interviewees ruminated over whether or not they should tell others about their diagnosis: “You wouldn’t say at a job interview, ‘yes, by the way, I have ADHD,’ because then you probably won’t get the job … If I meet a guy, how do I tell him about this?” (11). There was also concern that the diagnosis might be recorded in various places and used against them:

Still, it’s a threat, I’ve managed for 35 years and it feels like everything would suddenly be ruined now I have a diagnosis, that suddenly I might not be suitable for some stuff because no one knew this before … now suddenly it’s on file somewhere, somewhere in some database it says that I have the diagnosis of ADHD … it’s just that you don’t know how it can be twisted, that you have a diagnosis, how the authorities will treat it, even though it is a disability. (15)

##### The diagnosis as a self-fulfilling prophecy

The essence of this category was concern that an individual might not be as disabled as the ADHD diagnosis might imply; that a person’s possibilities in life might be underestimated because of the diagnosis, resulting in the diagnosis working as a self-fulfilling prophecy. Gaining knowledge of the difficulties associated with ADHD, during the diagnostic process, could result in a loss of self-confidence and the courage to try, for example, certain jobs or educational opportunities. One could lose one’s freedom and start living according to the diagnosis, letting the diagnosis guide one’s choices and thereby avoiding things which are considered difficult or impossible for people with ADHD. Certain behaviours were excused when related to ADHD, resulting in decreased motivation to change:

They told me it would be very difficult for me to have a management position … but what if I’m not like that, then it becomes an obstacle for me, because then I might believe it myself … You become very limited because you already find out when they are doing the workup and making the diagnosis that there are some tasks you can manage and others you can’t and some stuff you do and say may be because of it [ADHD], and then you believe it yourself and then you live based on that … I don’t really dare, no, but I’m not like everyone else, I have ADHD, I might not manage this because that’s what everyone around me and my psychologists are saying and it’s OK for me to be weird, I get to say strange stuff because I’m always excused anyway … They build my life in a way, in that they set limits and they give assistance that might or might not be needed. (20)

#### I did not get the follow-up and help I expected

The interviewees, represented in this category, described experiences of being left without the expected follow-up, help, and treatment after completion of the diagnostic process: “Then [after being diagnosed] they just dismissively sent me away with nothing, I was one of the crowd who was done … a failure, a betrayal from the healthcare system” (4). Their hopes of being offered professional information, advice, tools, means of assistance, medication, or conversational therapy were not fulfilled. They stressed the need for combining different treatment approaches: “It’s a huge handicap … I won’t get healthy just because I take a pill, I don’t think so, but you probably have to combine it … counselling and pills” (16).The experience of being met by ignorance from different helping authorities/institutions was also included in this category.

#### I wish I had been diagnosed earlier in life

This category included feelings of disappointment, frustration, anger, and sadness over not having been diagnosed earlier in life: “I think I felt sadness … lost time … how badly I was treated … it was, well, the whole thing, everything in those years [before the diagnosis] … ” (10). These feelings sprang from the belief that early diagnosis could have spared them suffering and provided possibilities of a better life through the understanding, help, treatment, and increased self-knowledge that followed diagnosis. Thoughts were expressed that an early diagnosis could have resulted in a different childhood: “ … if my mother had known what I know now, she would have treated me differently, just as I’m changing the way I treat my child” (3). Failures in school and working life could have been avoided:

You might have had a completely different life if it had been discovered earlier … if they had prescribed those drugs … if I had received these assistive devices … then I would have been able to get an education … I would have had a greater chance … because now you were just classified as the difficult pupil in the class. (19)

Relationships might have been saved:

I get a little angry that I didn’t get an answer earlier, I mean then maybe my life would have been different, maybe I would have lived together with the father of my children … the relationship ended because my mood just got worse and worse, so if I had received this diagnosis 10 years ago, maybe we would still be living together, peacefully and harmoniously, and the children might have been less stressed. If I drop a pot in the kitchen now, everyone becomes terrified. They think I threw it. (16)

Painful experiences of being taken advantage of and maltreated might have been avoided: “I could have been more on my guard … If I had been the way I am now [after diagnosis and treatment], I would probably not have suffered as badly back then as I did … ” (10).

Other difficult experiences that the interviewees believed might have been avoided included drug and alcohol abuse, criminality, depression, anxiety and fatigue syndrome, as well as the pain of being misunderstood and punished by others and blaming themselves for shortcomings due to lack of an alternative explanation: “Imagine if someone had figured out ten years ago that I had ADHD, then I’d have been spared all of this, wouldn’t have had to go to jail, maybe I’d have had a real job today … wouldn’t have had to live the life I’ve lived” (6).

The interviewees’ belief in the positive impact of early diagnosis seemed to be based on their having experienced pronounced improvement of their well-being and possibilities in life after being diagnosed:

I: Have you ever thought about what it would have been like if you hadn’t been worked up, diagnosed and treated?

IP: Yes, I have, I probably would have killed myself, actually. (21)

#### Being aware that ADHD is hereditary makes me see my parents and children differently

The knowledge of having a hereditary disorder made the interviewees wonder whether their close relatives, such as parents and children, suffered from the same condition. This awareness could increase their understanding of their own parents: “My mother has strong ADHD, I can say, though she hasn’t been diagnosed, and being diagnosed myself has helped me to understand her better” (3). It could also trigger grief over parents who had not been offered any help because they were never diagnosed: ” … I get very sad when I think about it, because I am mainly thinking about my mother, who has it, but doesn’t know it” (1). Some individuals also actively looked for ADHD symptoms in their own children, because they considered early diagnosis and help important in order to avoid the suffering they had experienced themselves prior to diagnosis:

IP: What should I think about my daughter’s behaviour, is there any way I can begin now to compensate for, for her then … I’m still not at all sure that she is in the same spectrum as I am, but I have to constantly be aware so that she doesn’t get into trouble.

I: But how do you think your knowledge about the diagnosis can help her?

IP: By perhaps not setting demands that are impossible for her to meet, which could have quite dramatic consequences for her self-confidence, by showing patience in areas where I normally wouldn’t. (1)

## Discussion

The positive consequences of being diagnosed with ADHD were dominant in this material. However, there was a rich and complex intra- and inter-individual variety of different experiences. Descriptions were focused on the diagnosis, on identity, and on life. The diagnosis was described as explaining a life history that was previously hard to understand, but the diagnosis was also questioned, both as a phenomenon and in relation to the individual (*the diagnosis* in focus). It was experienced as providing self-knowledge and increased value, but could also cause devaluation and raise questions of identity (*identity* in focus). It was described as bringing about a better life situation, but also as restricting possibilities and causing disappointment over lack of professional help. Sadness and anger were expressed in relation to a wish for earlier diagnosis that might have spared suffering. A changed view of relatives was described (*life* in focus). One possible explanation for the intra-individual variety is that an individual exists in different contexts, such as family, friends, school, the workplace, and different authorities; reactions to (and consequences of) an ADHD diagnosis can vary between these contexts. An individual’s ways of relating to these different contexts can result in a variety of seemingly incompatible experiences and perceptions.

Even though previous qualitative studies in the field have used different methods and foci, most of the experiences reported in the present study are supported by similar results from those studies, while some experiences, to our knowledge, have not been described before (e.g. “Others don’t think as badly of me anymore”, “I can help myself“, and “The diagnosis as a self-fulfilling prophecy”).

### Perceived positive consequences

The results showed that individuals experience major benefits from being diagnosed with ADHD as compared to the pre-diagnostic situation. All participants would have chosen to go through the neuropsychiatric evaluation that resulted in an ADHD diagnosis again, had they been undiagnosed at the time of the interview. This could be seen as their overall value judgement of being diagnosed with ADHD. The experience of the diagnosis can, however, be connected to, and influenced by, the experience of the diagnostic process. One of the interviews (18) was dominated by negative consequences, and we cannot rule out the possibility that it is the opinion of the neuropsychiatric evaluation that is reflected in the overall value judgement. Our general impression from the interviews was that the neuropsychiatric evaluation was regarded as an important learning situation, where the individuals got to know themselves better, as well as a situation where their experiences were validated.

All but two of the interviewees welcomed ADHD as a satisfying explanation for the difficulties and failures they had experienced, and for the feeling of being different. The diagnosis gave relief, as it brought an end to the feeling of confusion, the frustration of not understanding, and the energy-consuming search for an explanation. The same experience of life becoming more comprehensible through the diagnosis of ADHD was described by Young and co-workers [29] and by Fleischmann and Fleischmann [28]. About half of the individuals in the present study expressed another consequence of diagnosis, namely the gaining of important new knowledge about themselves, which sometimes made them assess their own behaviour more critically. All but two of our interviewees felt that the transition from being undiagnosed to being diagnosed gave them an increased value. ADHD was considered less detrimental to their identity than having poor character traits, because ADHD as a medical diagnosis is regarded, by both the individual and the surrounding world, as a legitimate reason for shortcomings. Misbehaviours would no longer be interpreted as actions carried out purposely or wilfully, but as symptoms of a medical disorder. Consequently, the individual’s self-worth would be protected. This post-diagnosis relocation of blame from the individual to the disorder has been reported in previous studies [28-30], as has the positive consequences for self-perception. Toner and co-workers [30] described effects of increasing acceptance, understanding, and tolerance towards oneself that precede the results of the present study. In addition, individuals in the present study not only reported that being diagnosed had important positive effects on how they saw and treated themselves, but also on how others saw and treated them. All but one of our interviewees reported that the diagnosis of ADHD allowed them to access help that could have a positive impact on their lives. They noted that it was hard to get help for difficulties which were not diagnostically framed. It was considered better to have ADHD than to be “difficult”, because the consequences differ; you receive help instead of, in some cases, being “punished”. The same theme of life becoming more manageable after being diagnosed, through medication and coping strategies, also emerged in earlier studies [28-30].

The category “I can help myself” was a key result, which, to our knowledge, has not been described in previous studies. Within this category, the diagnosis can be seen as a potential resource in treatment work. Following the ADHD diagnosis, certain behaviours were revised, no longer considered parts of oneself, but re-characterized as symptoms of a disorder, and thus regarded as wrong and unwanted. In this way the diagnosis in itself seemed to serve as a strong motivating factor for change, enabling individuals to mobilize their own resources and change their behaviour, resulting in increased well-being and improved functioning in everyday life.

Our results suggest that many individuals diagnosed as adults have a past life history of suffering, not only related to ADHD symptoms but also to the absence of a satisfying explanation for the difficulties they have experienced. This results in confusion and frustration; being misunderstood, negatively judged, and badly treated; not receiving adequate help; and blaming oneself for failures in different areas of life, resulting in a low self-worth. Whether or not these individuals would have had a more positive life history had they been diagnosed during childhood is impossible to determine in retrospect, but this was a commonly expressed view in the material. More than half of the interviewees expressed a wish for earlier diagnosis that could have spared suffering and made life turn out differently, in line with findings by others [26,29,30].

ADHD, both diagnosed and undiagnosed, has the potential to interfere with and affect a person’s identity in a number of different ways. It is clear that the ADHD symptoms border on what is considered one’s personality: “ADHD is part of my personality” (3). Some of the interviewees had not only come to terms with having ADHD, but did not want to be without it. They believed that certain parts of the ADHD symptomatology contributed in an important positive way to their personality and life. The perception of ADHD as an advantage was also described by Fleischmann and Fleischmann [28].

### Perceived negative consequences

Despite the above positive descriptions, about half of the participants also expressed substantial negative consequences of being diagnosed with ADHD. It seems important for professionals to be aware that these negative aspects exist, so that individuals can be offered help to deal with them. The category “The diagnosis causes decreased value” is a reminder that the problem of stigmatization is real and needs to be addressed. In the studies mentioned above by Young and co-workers [29] and Toner and co-workers [30], participants reported an awareness of the stigma associated with ADHD. In the present study, apart from experiences of devaluation, the main negative consequences were fear of the diagnosis becoming a self-fulfilling prophecy, through loss of self-confidence, courage, and motivation, and fear of discrimination. The diagnosis was perceived as an obstacle, limiting their possibilities in different situations in life. The diagnosis could also raise questions concerning “who I am” and impose on identity in a number of ways. However, the overall impression is that these negative effects were more than compensated by the positive consequences.

### Limitations

Although our study revealed both positive and negative experiences, and the interview stage was not concluded until no new experiences emerged, it is impossible to ensure that the full variation of experiences in a population is covered. Moreover, even though some experiences might be the same, the results cannot automatically be generalized to other populations. The individuals were recruited from the same specialized clinic, they had all been motivated to go through a neuropsychiatric evaluation, and the group was relatively homogenous in terms of age and time since diagnosis. In addition, it was necessary to exclude individuals with severe comorbid conditions in order to isolate the phenomenon. Finally, the experiences found here undoubtedly also reflect the socio-cultural context in which the participants live. Therefore, individuals who, for example, are more reluctant to seek help, have a different comorbid setup, or live in a society with different views on ADHD might have different experiences.

## Conclusion

The dominance of perceived positive consequences of being diagnosed with ADHD support that adults who may have undiagnosed ADHD should be offered a neuropsychiatric evaluation. Furthermore, the lack of early detection seems to carry potential negative effects. Our interviewees highlighted the importance of the transition between the diagnostic and treatment processes, as they expressed experiences of abandonment after the neuropsychiatric evaluation was complete. Diagnosis of ADHD should be followed by a broad offer of treatment and follow-up characterized by an overall view of the individual’s life situation, self-perception and health care needs, and not merely focused on medication.

It is especially important for professionals to be aware of potential negative consequences of being diagnosed. Patients’ experiences can affect well-being and interfere with different treatments. Some positive experiences can be considered resources (e.g. “I can help myself”) while negative experiences might create obstacles (e.g. “The diagnosis causes decreased value”). Knowing a person’s combination of experiences can improve cooperation and facilitate planning of treatment to meet individual needs. Knowledge of the rich palette of different experiences can facilitate introduction of alternative or complementary ways of thinking.

Finally, using patients’ perspectives on diagnoses may be a further step in validating psychiatric diagnoses.
